# Global Disease Outbreaks Associated with the 2015–2016 El Niño Event

**DOI:** 10.1038/s41598-018-38034-z

**Published:** 2019-02-13

**Authors:** Assaf Anyamba, Jean-Paul Chretien, Seth C. Britch, Radina P. Soebiyanto, Jennifer L. Small, Rikke Jepsen, Brett M. Forshey, Jose L. Sanchez, Ryan D. Smith, Ryan Harris, Compton J. Tucker, William B. Karesh, Kenneth J. Linthicum

**Affiliations:** 1 Universities Space Research Association, Columbia, Maryland USA; 2 NASA Goddard Space Flight Center, Biospheric Sciences Laboratory, Greenbelt, Maryland USA; 3 Department of Defense, Armed Forces Health Surveillance Branch, Silver Spring, Maryland USA; 4 USDA-Agricultural Research Service Center for Medical, Agricultural, and Veterinary Entomology, Gainesville, Florida USA; 5 Science Systems and Applications, Inc., Lanham, Maryland USA; 6 Cherokee Nation Technology Solutions, Silver Spring, Maryland, USA; 7 United States Air Force, 14th Weather Squadron - DoD Climate Services, Asheville, North Carolina USA; 8 EcoHealth Alliance, New York, New York USA; 9 Present Address: National Center for Medical Intelligence, Fort Detrick, Maryland USA; 10 Present Address: Interstate Commission on the Potomac River Basin, Rockville, Maryland USA

**Keywords:** Ecology, Climate sciences, Biogeography, Environmental impact, Infectious diseases

## Abstract

Interannual climate variability patterns associated with the El Niño-Southern Oscillation phenomenon result in climate and environmental anomaly conditions in specific regions worldwide that directly favor outbreaks and/or amplification of variety of diseases of public health concern including chikungunya, hantavirus, Rift Valley fever, cholera, plague, and Zika. We analyzed patterns of some disease outbreaks during the strong 2015–2016 El Niño event in relation to climate anomalies derived from satellite measurements. Disease outbreaks in multiple El Niño-connected regions worldwide (including Southeast Asia, Tanzania, western US, and Brazil) followed shifts in rainfall, temperature, and vegetation in which both drought and flooding occurred in excess (14–81% precipitation departures from normal). These shifts favored ecological conditions appropriate for pathogens and their vectors to emerge and propagate clusters of diseases activity in these regions. Our analysis indicates that intensity of disease activity in some ENSO-teleconnected regions were approximately 2.5–28% higher during years with El Niño events than those without. Plague in Colorado and New Mexico as well as cholera in Tanzania were significantly associated with above normal rainfall (p < 0.05); while dengue in Brazil and southeast Asia were significantly associated with above normal land surface temperature (p < 0.05). Routine and ongoing global satellite monitoring of key climate variable anomalies calibrated to specific regions could identify regions at risk for emergence and propagation of disease vectors. Such information can provide sufficient lead-time for outbreak prevention and potentially reduce the burden and spread of ecologically coupled diseases.

## Introduction

Fluctuations in weather and climate at various time scales regulates the functioning of ecosystems which in turn affects abundance of plant and animal life, in particular populations of an array of disease vector insects which influence the potential for occurrence of disease epidemics and epizootics^1,2^. On an interannual time scale, the El Niño-Southern Oscillation (ENSO) phenomenon has profound impacts on global climate and weather anomaly patterns, often defining major peaks in spatial and temporal dimensions of drought and flood conditions^3–6^. These extremes in precipitation and temperature resulting from ENSO events are now known to be the background drivers of a range of vector- and water-borne diseases, and coral diseases, whose peaks in activity coincide, lag, or follow precipitation and temperature departures from normal^5–11^. The persistence of extreme conditions of either temperature or precipitation impacts the ecology and habitat size of different vectors; vector population growth rates and dynamics, distribution, and seasonality; replication and extrinsic incubation of a virus in the vector; and virus transmission patterns and seasonality^12–15^.

Although ENSO is primarily a tropical phenomenon initiated by changes in the coupling between the equatorial tropical Pacific Ocean sea surface temperatures and the atmosphere, it has far reaching consequences, or teleconnections, on global atmospheric circulation and impacts that extend beyond the tropics, affecting the extra-tropics especially parts of North America^3–6^. Since ENSO impacts are expressed via regional teleconnections worldwide as floods or droughts^3–5^, the consequence is to produce various disease epizootics and epidemics in such regions^7–10,12,13^ (Table 1). Historically, disease epidemics and epizootics have followed climatic departures from norms in regions influenced by ENSO. This is due to the development of favorable ecological conditions under which arthropod and rodent vectors of human and livestock pathogens emerge in large numbers with enhanced survival and vectorial capacity^16^, thereby greatly increasing disease transmission risk^10,12,13^. In some regions, ENSO events are associated with amplification of endemic diseases such as dengue^17,18^, malaria^19^, cholera^12^, and hantavirus^20^ (Table 1). Whereas in others, ENSO events act as a trigger for disease outbreaks such as in East Africa where an ENSO warm event precedes Rift Valley fever outbreaks^21^, or in tropical highland and drylands regions where El Niño directly drives malaria epidemics^22^. ENSO events are also associated with an array of other major public health impacts such as respiratory illnesses from drought-induced wildfire smoke, flood-induced epidemics of water-borne illnesses, and nutritional deficiencies due to crop failures^2,10,11^ (Table 1).

**Table 1:** *El Niño*-Associated Disease Transmission Enhancement in Human/Livestock Populations: Examples.

Disease	Region	Possible *El Niño* Effects on Disease Dynamics
Cholera	Africa^52,80^: Great Lakes region; Asia^10,51,81–83^ South Asia: Bangladesh, India (coastal), Sri Lanka;	Warmer water temperatures promote bacteria proliferation; flooding causes contamination of water sources, and may increase susceptibility to infection via stress.
Dengue/chikungunya	Asia/Pacific^10,17,84–87^: Indonesia, Thailand, Pacific Islands, Australia (Queensland); North America^88–90^: Mexico, United States (southern tier); Northern South America: Caribbean Islands^10,91^, French Guiana, Suriname	Dry conditions: Peri-domestic water storage promotes *Aedes aegypti* mosquito vector breeding; elevated temperatures reduce the virus extrinsic incubation period in *Ae*. *aegypti* and *Ae*. *albopictus* vectors; warm, dry conditions may promote vegetation patterns favorable for vector development. Wet conditions: Elevated rainfall promotes *Ae*. *aegypti* and *Ae*. *albopictus* breeding.
Hantavirus infection	Asia^92–95^: China (eastern; hemorrhagic fever with renal syndrome); North America^20^: United States (southwestern; hantavirus pulmonary syndrome)	Elevated rainfall increases food availability for rodent reservoirs (vegetation), which expands rodent populations and may promote contact with humans.
Malaria	South Asia^10,96,97^: India, Sri Lanka, Bangladesh; South America^10,98–100^: Colombia, French Guiana, Guyana, Peru (coastal), Venezuela, Africa: Great Lakes Region	Elevated rainfall promotes *Anopheles* mosquito vector breeding and survival, and vectorial capacity.
Plague	Africa^101^: Madagascar; North America^102^: United States (western)	Heavy rains increase food availability for populations of susceptible rodents; cooler temperatures may increase infectious flea abundance.
Rift Valley fever	Africa^7,13^: East Africa	Flooding of dry mosquito vector habitats promotes hatching of (transovarially-) infected eggs, and vector breeding and survival.
Respiratory illness	Asia^103,104^: Southeast Asia/Indonesia	Drought may contribute to forest fires, which cause air pollution that may increase risk of respiratory infection.
Ross River virus disease	Asia^10,105^: Australia (Queensland/Murray-Darling River region)	Warm conditions may increase mosquito vector longevity, and thereby vectorial capacity.

In this paper, we describe collaborative efforts for the first time by the National Aeronautical and Space Administration (NASA), the US Department of Agriculture (USDA), and the Department of Defense (DoD) to leverage a variety of earth orbiting satellite data sources and ENSO monitoring and mapping to protect public and veterinary health worldwide. Throughout the 2014–2016 period, this interagency group systematically monitored the development of ENSO-induced environmental conditions conducive to elevated disease transmission risk. The exceptionally strong ENSO event of 2015–2016 generated excess rainfall and flooding, drought, and temperature extremes that created ecological conditions potentially favoring disease transmission in affected regions worldwide. As the extents of these ENSO-affected regions were determined, the interagency group developed and disseminated disease outbreak warnings calibrated by seasonal forecast information and systematically monitored disease outbreaks in affected regions worldwide to further refine the relationship between ENSO and public health disease risks. Table 1 shows a summary of our *a priori*, expected disease outbreaks following the ENSO event, regions of focus, as well as associated climate drivers and/or amplifiers underlying the disease outbreak activity.

## Results

### ENSO-induced anomalies in weather and environmental conditions worldwide

**Figure 1:**
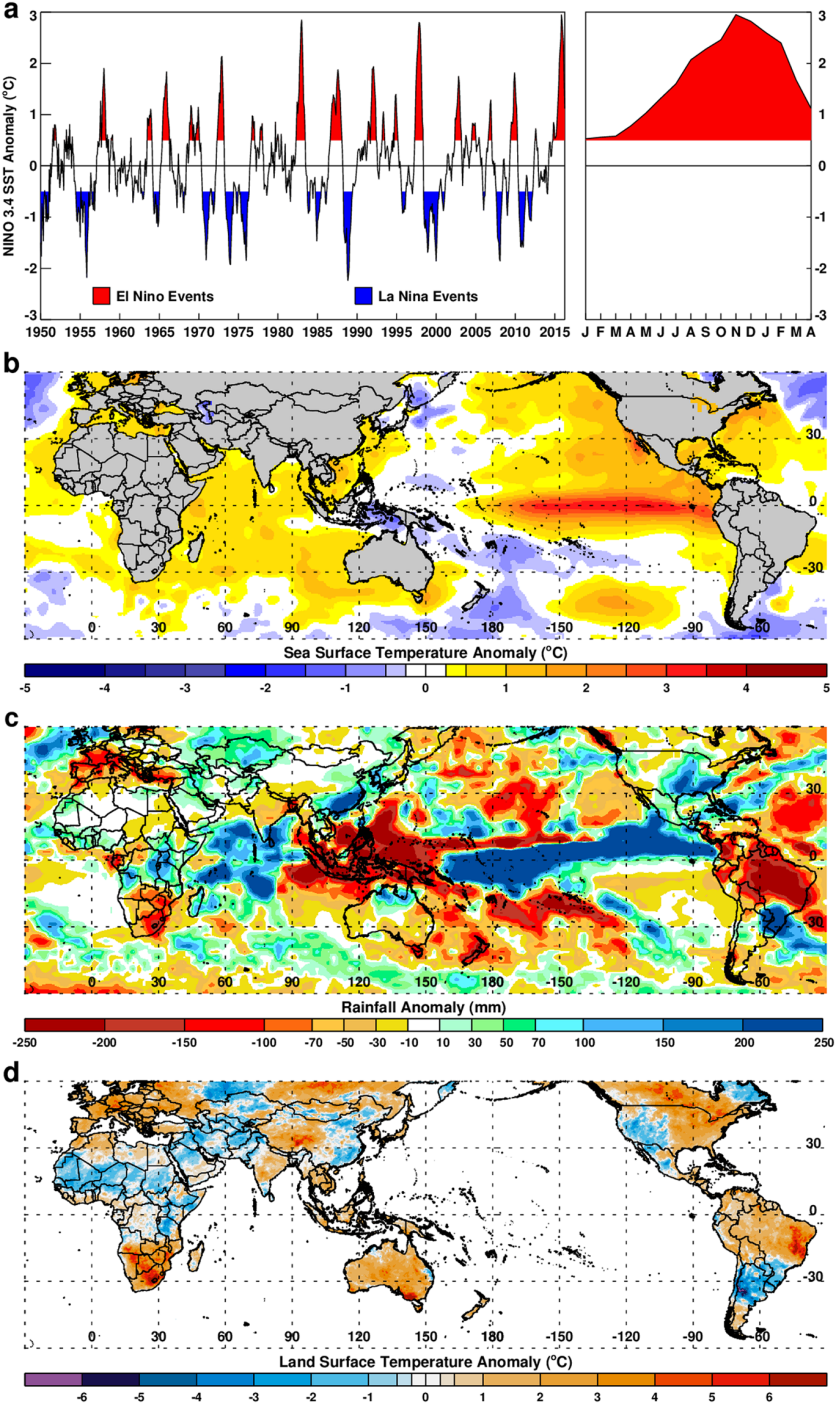
Climate anomalies during the 2015–2016 ENSO event: (**a**) 1950–2016 NINO 3.4 sea surface temperature (SST) anomalies showing periods of *El Niño* and *La Niña* events defined by +0.5/−0.5 SST thresholds, and 2015–2016 *El Niño* SST anomaly values by month in red on right panel. (**b**) December-February 2015/16 global mean SST anomalies during the peak ENSO season. (**c**) October-December 2015 cumulative rainfall anomalies towards the ENSO peak phase and (**d**) mean land surface temperature (LST) anomalies for October-December 2015. Anomalies in rainfall and LSTs highlight several ENSO-linked regions including southeast United States, northeast Brazil, eastern equatorial Africa, southern Africa, and Southeast Asia. This figure was created using Interactive Data Language (IDL) software (version 8.6.0) (www.harrisgeospatial.com/SoftwareTechnology/IDL.aspx).

The 2015–2016 ENSO is ranked among the top three since 1950 according to National Oceanic and Atmospheric Administration (NOAA) evaluations^23^. The first indications of strong ENSO conditions emerged in spring 2015, having started to develop in the late fall on 2014, as demonstrated by increasingly high sea surface temperature (SST) in the NINO 3.4 region at the time (Fig. 1a – right panel, see Materials and Methods for NINO 3.4 region definition). Measurements of SST exceeding a threshold of +0.5 °C from the long-term mean in the NINO 3.4 region have historically served as a reliable sentinel of El Nino conditions^24^. This higher-than-normal SST anomaly reached its peak conditions in December 2015-February 2016 (Fig. 1a right panel and Fig. 1b). This event quickly affected ENSO-linked regions worldwide with extreme rainfall conditions that subsequently brought floods or droughts which persisted into fall and winter months, as follows. Significant above-normal rainfall anomalies were recorded during spring and early summer of 2015 in the central and southwestern US (+30 to +250 mm above normal in May–July; Fig. 1c and Table 2) and eastern India-Bangladesh (~+200 mm above normal in May–August; Fig. 1c and Supplementary Fig. S2). Above-normal rainfall anomalies were also observed during fall and winter (October–December) of 2015 in southern Brazil-Uruguay-Argentina region (~+250 mm; Supplementary Fig. S3) and over central to eastern equatorial Pacific, western Sahel, and eastern equatorial Africa regions (~+50 to +250 mm; Supplementary Fig. S3). Conversely, northern South America, Central America-Caribbean Islands, Southeast Asia, Gulf of Guinea coast, and Southern Africa experienced below-normal rainfall cumulatively with deficits up to ~−200 mm, indicating severe drought, during the October–December season (see Supplementary Fig. S3). These rainfall conditions occurred in the aforementioned regions during the respective critical growing seasons with large departures from the norms (~14–80% of the long-term mean, Table S1).

**Table 2:** Seasonal rainfall, long-term means and anomalies for various disease outbreaks in regions highlighted in Fig. 2a during the 2015–2016 ENSO warm event.

Region	Season	Seasonal Total (mm)	Seasonal Mean (mm)	Cumulative Anomaly (mm)	Anomaly (%)
Western US 31N–42N, 109W–102W	MJJ 2015	247.99	136.96	111.03	81.06
NE Brazil 15S–2.5S, 45W–35W	SOND 2015	62.33	223.97	−161.64	−72.17
Tanzania 10S–2.5S, 30E–37.5E	ONDJ 2015/2016	609.98	385.81	224.17	58.10
SE Asia 10S–7.5N, 97.5E–117.5E	ONDJF 2015/2016	1085.38	1265.43	−180.05	−14.23

These ENSO-driven anomalous rainfall conditions further propelled anomalies in two other key environmental parameters, land surface temperature (LSTs) (Fig. 1d) and vegetation as represented by the normalized difference vegetation index (NDVI) (Supplementary Fig. S4). Between October to December 2015, anomalously high LSTs were observed in Southeast Asia, Australia, Southern Africa, Brazil, and northernmost South America (Fig. 1d). Conversely, anomalously cool LSTs were observed during this period in equatorial East Africa, Southern Brazil, Uruguay, Argentina, and the western and southwestern United States, especially Texas and the intermountain states (Fig. 1d). The coupling of anomalous rainfall, temperature, and vegetation development, whether above-normal or below-normal, has been found to create a suite of habitat attributes conducive to unusually high pathogen-vector emergence and survival, and thus increased vectorial capacity and risk of disease transmission to humans and livestock^1,2,25–29^.

In response to observing the ENSO-driven weather conditions, we used seasonal rainfall forecast capabilities^30^ (Supplementary Fig. S1) and monitoring to issue early warnings for possible outbreaks of various climate-sensitive diseases, including: (1) Rift Valley fever, malaria, and cholera in East Africa, (2) malaria in Peru and Colombia, (3) cholera and malaria in Bangladesh and coastal India, (4) hantavirus pulmonary syndrome and plague in the US west and southwest, and (5) dengue fever and respiratory illnesses due to drought conditions and large-scale forest fires in Southeast Asia and northeastern Brazil^31^. Additionally, there was increased risk of chikungunya and Zika outbreaks in northeastern Brazil. Early warning alerts were issued on a monthly or quarterly basis beginning in the fall of 2014 through 2016 period by an interagency group composed of NASA Goddard Space Flight Center, USDA Agricultural Research Service Center for Medical, Agricultural, and Veterinary Entomology, and Defense Health Agency’s Armed Forces Health Surveillance Center. Alerts were issued when elevated conditions for certain anomalies were projected in specific regions and confirmed by continuous monitoring^31^. This information was shared with concerned federal interagency partners and international collaborators including World Health Organization (WHO) Health Emergencies Programme, Food and Agriculture Organization of the United Nations (FAO)‘s Animal Health Service (AGAH) - Emergency Prevention System for Transboundary Animal and Plant Pests and Diseases (EMPRES) and World Organization for Animal Health (OIE)’s Animal Health and Information Department as periodic update reports. Information distribution and release was also coordinated by the interagency Pandemic Prediction and Forecasting Science & Technology (PPFS&T) Working Group^32^.

**Figure 2:**
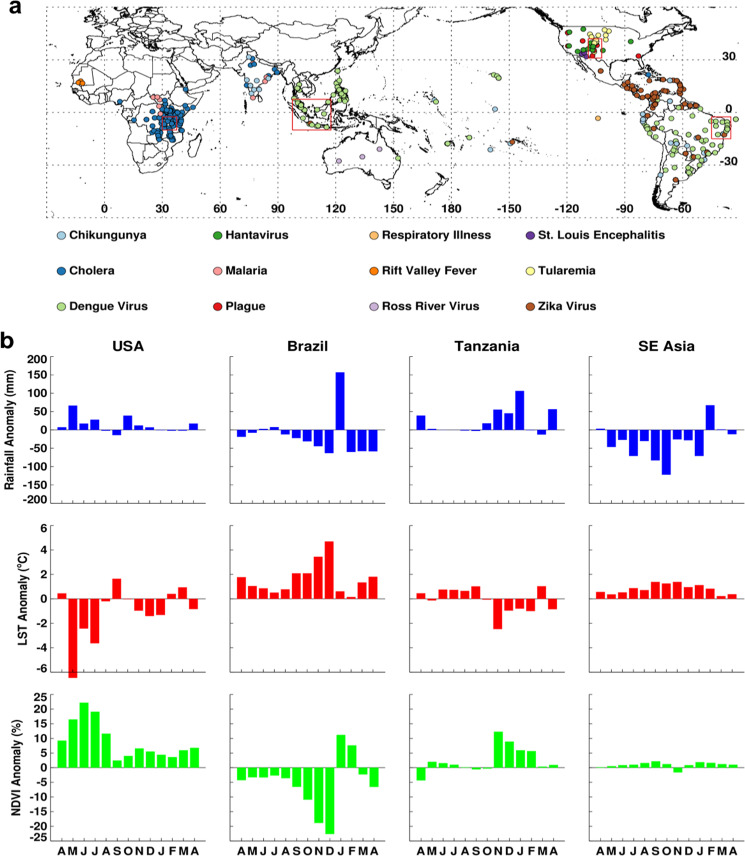
Geographic distribution of various disease activity worldwide (between April 2015–March 2016) compiled from various sources (**a**) and time series profiles of climate variables (**b**) for each box in (**a**). Persistence of anomaly conditions of precipitation, land surface temperature, and normalized difference vegetation index in (**b**) created conditions for the emergence of vectors and outbreaks of diseases for United States, Brazil, Tanzania, and Southeast Asia focal regions in (**a**). This figure was created using Interactive Data Language (IDL) software (version 8.6.0) (www.harrisgeospatial.com/SoftwareTechnology/IDL.aspx).

Following the early warning alerts, we systematically monitored and mapped the aforementioned climate-sensitive disease occurrences in ENSO-linked regions from 2015 to April 2016 (Fig. 2a) as reported through ProMED. The geographic distribution of various disease activity shown in Fig. 2a clearly illustrated the tendency of outbreaks of several diseases to cluster in the focal ENSO teleconnection regions, where in other years these disease distributions are either more widespread or not large enough that they are not reported (Fig. S4). The western and southwestern states of the US (New Mexico, Arizona, Colorado, Utah, Texas, and California) suffered outbreaks of hantavirus pulmonary syndrome and plague in 2015–2016 (Fig. 2a). Many locations in California and central and southern US regions also reported increased cases of West Nile fever (Supplementary Fig. S5). During this time, above normal heavy rainfall – followed by increased vegetation – was observed in most continental central and western United States (Supplementary Figs S2 and S3), potentially elevating *Culex* mosquito populations. There were also several ENSO-teleconnected US areas reporting cases of otherwise uncommon diseases in this period. For example, the first isolations of St. Louis encephalitis virus (SLEV) in mosquitoes in Arizona and California since 2007 (an ENSO year) and 2003, respectively, were reported in 2015. The first human cases of St. Louis encephalitis in Arizona since 2006 were also reported during 2015^27^. The impact of 2015 ENSO-driven rainfall on elevating populations of *Culex tarsalis* and *Culex quinquefasciatus* mosquito vectors of SLEV may have played a role in these observed increases in detection and transmission of SLEV. Elevated rainfall linked to 2015–2016 ENSO activity may have also caused spikes and clusters of Tularemia - an endemic but otherwise rare disease in North America – in Wyoming, Colorado, and North and South Dakota, by increasing tick and deer fly vector as well as rodent and lagomorph reservoir populations (Fig. 2a and Supplementary Fig. S6). In 2015, CDC reported an increase in human cases of Tularemia in the United States with a total of 100 cases between January–September 2015, comparable to the total number of cases between 2000–2010 (125 cases)^33^. Colorado experienced the highest increase in cases per 100,000 populations from the annual mean (~11-fold increase), followed by Wyoming and Arizona (Supplementary Fig. S6).

Many areas of South and Central America and the Caribbean Islands had coincident outbreaks of Zika, dengue fever, and chikungunya in 2015–2016 (Fig. 2a and Supplementary Fig. S3). Severe ENSO-linked drought conditions and elevated temperatures in this region (Figs 1d and 2b Brazil, Supplementary Fig. S3) increased risk for these vector-borne infections as well as respiratory illnesses. While the introduction of Zika virus into this region may be unrelated to the 2015–2016 weather conditions, the epidemic of Zika, transmitted by *Aedes aegypti* and possibly *Aedes albopictus* mosquito vectors over such a wide geographic range was likely facilitated and amplified by the persistence of above-normal temperatures and drought conditions over this region^2,25,31,34^, enabling the virus to spread rapidly in ~31 countries in the Western Hemisphere^35^. Severe drought conditions and above-normal temperatures are a common feature of the northern South America region during warm ENSO events^3–5^ and have been associated with increased dengue virus transmission and respiratory illnesses^10,25^.

The Pacific Islands including Papua New Guinea, Tahiti, Hawaii, American Samoa, Cook Islands, Fiji Islands, French Polynesia, Marshall Islands, and Kiribati had outbreaks of dengue fever and chikungunya which also coincided with severe drought conditions (Supplementary Fig. S7). This drought was a result of the shift in the center of maximum precipitation from the western Pacific equator towards the eastern Pacific under El Niño conditions (Supplementary Fig. S2). In some instances, such as dengue fever in Hawaii where local dengue transmission by mosquitoes occurred but did not occur for Zika, outbreaks were attributed to introductions from infected travelers from endemic regions. This demonstrates trade, travel, and transport networks serve as major avenues for introductions of various diseases beyond their endemic region during periods of large epidemics such as observed during the 2015–2016 ENSO event. Although it is not trivial to unravel the role of mobilization (trade, travel, and transport networks) from climate variability on disease outbreaks, areas with high tourism and trade could possibly be affected by the amplification of climate-sensitive diseases independent of regions affected by ENSO events.

**Figure 3:**
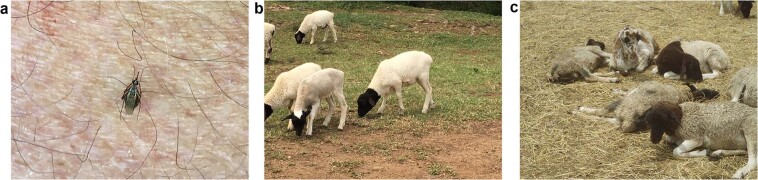
*Aedes mcintoshi* Rift Valley fever virus reservoir mosquito at a farm in Ruiru, near Nairobi, Kenya (left-**a**) in January, 2016, produced by anomalously heavy rainfall in the presence of healthy sheep (center-**b**) unlike in January, 2007 (right-**c**) when the farm lost ~80% of its sheep population. Early warning and early vaccination prevented transmission of Rift Valley fever 2016 on this farm (*Photo Credits: KJ Linthicum and A*. *Anyamba*).

Western Sahel experienced wetter-than-normal rainfall season in October–December 2015 that brought above-normal vegetation conditions (Supplementary Fig. S3). Shortly thereafter, Rift Valley fever outbreaks occurred in Mauritania (between June 2015–March 2016) and increased malaria cases were reported in Sudan, Southern Sudan, Uganda, Kenya, Somalia, and Tanzania^36^ (Fig. 2a). A large regional cluster of cholera cases was observed in East Africa (Kenya, ~13,299 cases, Tanzania, ~20,715 cases)^36^ following heavy rainfall and flooding (Fig. 2). This epidemic of Cholera got amplified to cover a much broader region continued through 2017 (Fig. S4C). Unlike during past significant ENSO events, no epidemics/epizootics of Rift Valley fever were reported in East Africa in the 2015–2016 period; with only a focal outbreak reported in southwestern Uganda, an area outside of the typical Rift Valley fever epizootic zone. Our field surveillance in early 2016 indicated that although abundant *Aedes mcintoshi* mosquitoes, the primary reservoir vector of Rift Valley fever emerged from multiple persistent floodwater sites, no significant outbreaks were detected during this period. Proactive measures to conduct mass vaccination campaigns of livestock in areas at risk of elevated rainfall had taken place following our warnings issued in the Rift Valley Fever Monitor^37^ in early 2015. The warning and timely vaccination likely averted epizootics and epidemics, unlike in 2006–2007 when response to an imminent outbreak was delayed^38^ (Fig. 3).

In the Southeast Asia region, we observed increased incidence of dengue and chikungunya during 2015–2016 (Fig. 2a). Throughout this period (April 2015–March 2016), rainfall was anomalously low in Southeast Asia (Fig. 2b), which may have contributed to the increased dengue and chikungunya activity^39^. In addition to vector-borne diseases, there was also an increase in respiratory illnesses related to smoke produced from uncontrolled burning of tropical forests due to extreme high temperatures and persistent drought^35^. Cholera, malaria, and chikungunya outbreaks were reported in India and Bangladesh (Fig. 2a) as has been observed during previous ENSO events^12^.

### Disease incidents during 2015–2016 ENSO period

To illustrate the associations between ENSO-forced climate anomalies with disease outbreaks, we have selected 4 diseases in 4 regions. These are hantavirus and plague in the intermountain western region of the United States (Colorado and New Mexico), cholera in Tanzania, and dengue in Brazil and Southeast Asia. For this analysis we compiled disease outbreak information from ProMED reports^40^ since 1996 to 2016 in order contextualize disease activity during the 2015/2016 ENSO event over the recent historical period. In addition, for this analysis we compiled annual dengue cases in Brazil^41^ and annual cholera cases Tanzania^42^.

#### Plague and Hantavirus in Colorado and New Mexico (United States)

Both hantavirus pulmonary syndrome (HPS) and plague are common to southwestern United States region, in an area shared by Arizona, New Mexico, Colorado, and Utah known as “The Four Corners^43,44^”. Sin Nombre virus which causes HPS is transmitted to humans by rodents, such as mice and rats. Plague, on the other hand, is a disease caused by the bacterium *Yersinia pestis* and spread to humans when one is bitten by plague-infectious flea vectors or when one comes in contact with plague-infected hosts. Rodents and other small mammal species are a common factor for hantavirus and plague maintenance and amplification in the western United States^45,46^. In both instances, unless infected persons are quickly treated with antibiotics, the diseases are fatal to humans. A number of studies have shown that during periods when there is an increase in the number of cases of both plague and hantavirus, climatic conditions are favorable to both hosts and vectors^46,47^. Such conditions are exemplified by above-normal precipitation and mild temperatures at regional to large scales, resulting in a tendency of outbreak clusters concentrated in this region. Such periods of above-normal rainfall and cooler temperatures in this semi-arid region are associated with the occurrence of the warm phase ENSO which typically increases food resource availability, cascading into an increase in populations of both hosts (mice, rodents) and flea vectors^48^.

**Figure 4:**
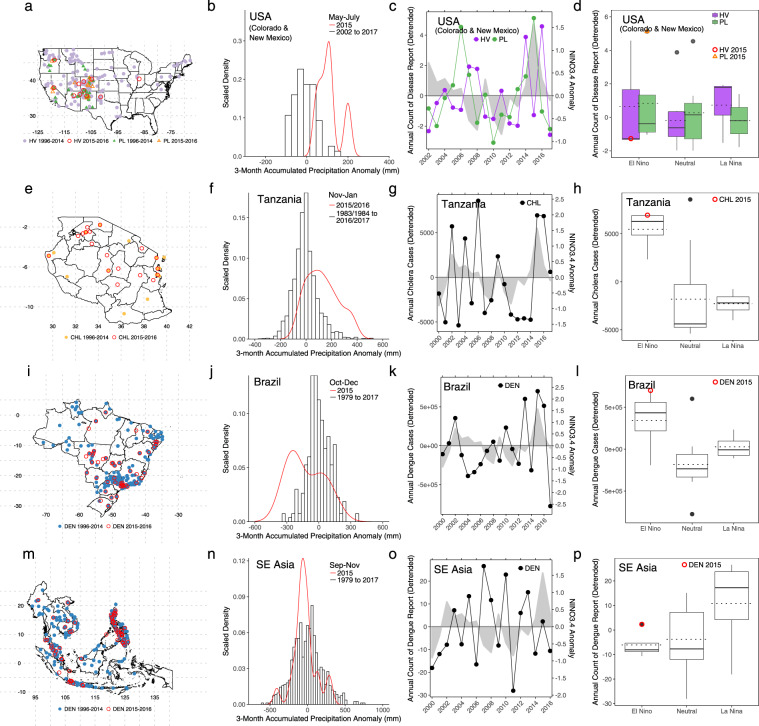
Selected regional disease outbreaks and climate conditions for hantavirus (HV) and plague (PL) in the United States (**a**–**d**); cholera (CHL) in Tanzania (**e**–**h**); dengue (DEN) in Brazil (**i**–**l**); dengue (DEN) in Southeast Asia (**m**–**p**). Maps in the first column show the locations of reported disease occurrences during April 2015 to May 2016 *El Niño* event, overlaid on the locations of the same diseases occurring between 1996 and 2014. Histograms in the second column show rainfall anomaly distributions for locations with reported disease occurrences during the specified season in the 2015/2016 *El Niño* year. Time series plots in the third column represent each disease intensity over the years while the shaded plot denote annual NINO3.4 anomaly. Boxplots in the fourth column show the distribution of each disease intensity as categorized by the ENSO events. Here solid black lines represent the median value, dotted lines the mean value, and the circles are the disease intensity during 2015/2016 *El Niño* year. This figure was created using R software (version 3.4.1)^79^.

During the 2015/2016 El Nino event (April 2015–March 2016), plague and hantavirus were more predominantly reported in the Four Corners region compared to previous years (Fig. 4a and Supplementary Fig. S4). Although plague and hantavirus are historically more prevalent in this region, there were no reported cases (through ProMED) in the northwestern areas such as Wyoming, Montana, and Washington where cases had typically been reported in previous years. Washington is the state with the 5^th^ highest cumulative hantavirus cases in the United States^49^ and we observed that during May–July 2015 (periods when hantavirus and plague typically occurred) rainfall was anomalously low (Supplementary Fig. S2) with a mean of 58.2 mm below normal across the state. The dry conditions were not favorable for the hosts to amplify the disease propagation in humans. In both Colorado and New Mexico, where plague and hantavirus were more frequently reported during the 2015/2016 El Niño event, rainfall was anomalously high between May–July 2015 (Supplementary Fig. S2). In locations where hantavirus and plague were reported in May–July 2015 we observed that rainfall in these locations was anomalously high (Fig. 4b) with a mean anomaly of 135.58 mm above normal. During El Niño events the southern and southwestern region of the United States tends to be wetter than normal, while regions to the north and northwest tend to be drier, thus creating conditions for clustering of plague and hanta virus disease activity in the Four Corners region.

As a proxy for disease intensity, we tallied the number of times that each hantavirus and plague case in both Colorado and New Mexico were reported in ProMED each year between 2002–2016 (Fig. 4c). After detrending the time series, we observed that plague intensity was highest in 2015 (at similar level to 2006), but not for hantavirus although it peaked in 2016. These observations are consistent with the CDC annual case counts for the entire United States^49,50^. Note that the derived ProMED and reported CDC counts were based on calendar year, whereas El Niño periods span April to March of the following year. Disease outbreak activity in response to an El Niño event often lags precipitation anomaly conditions, therefore it not a surprise that we observe increases in the disease counts the following calendar year after peak ENSO conditions.

The detrended intensity of plague activity is typically higher during El Niño years (Fig. 4d): approximately 28% higher than the mean intensity in the neutral years. In 2015, the intensity was at its highest, ~25% more than the mean intensity during other El Niño years. We further found that higher plague annual intensity was associated with higher rainfall anomaly (*P* < 0.05), but we did not find any significant association with LST anomaly (Table 3) even though lower-than-normal LSTs were observed during this period as would be expected during such an El Niño event. For hantavirus, the 2015 intensity was lower than the mean intensity during both El Niño years (11.88% lower) and neutral years (1.89% lower) (Fig. 4d). Consequently, we did not find any significant association between hantavirus annual intensity and both rainfall and LST anomalies during this period (Table 3) even though climate anomaly patterns during 2015–2016 were conducive for increase in hantavirus activity (Fig. 1c,d).

**Table 3:** Regression results.

Region	Disease	Dependent variable	Estimated Coefficient (95% Confidence Interval)		Adjusted R^2^
			Annual Rainfall Anomaly	Annual LST Anomaly	
Colorado & New Mexico	Hantavirus	Annual count of reports	−0.39 (−1.34, 0.56)	−0.54 (−1.32, 0.23)	0.15
Colorado & New Mexico	Plague	Annual count of reports	0.90 (0.01, 1.79)*	0.26 (−0.46, 0.99)	0.29
Tanzania	Cholera	Annual count of cases	0.79 (0.23, 1.35)*	0.15 (−0.36, 0.68)	0.32
Brazil & SE Asia	Dengue	Annual count of cases^a^ and reports^b^	0.27 (−0.08, 0.61)	0.52 (0.12, 0.92)*	0.53

* indicates significance at p < 0.05; ^a^for Brazil; ^b^for SE Asia region.

Our analysis in this region indicated the ENSO-induced above-normal rainfall was associated with plague and hantavirus. Although we did not find significant association between hantavirus and rainfall (Table 3), we observed increased hantavirus intensity during 2016 (Fig. 4c) that could potentially due to the ENSO-induced above-normal rainfall at the beginning of 2016 (Fig. 2b). Because the disease intensity was aggregated based on calendar year, the association with rainfall may not be derived at this temporal resolution. The ENSO-induced above-normal rainfall in this region resulted in abundant vegetation increasing food resources, which are factors known to elevate hantavirus and plague rodent vector populations^26^.

#### Cholera in Tanzania

While cholera is not a major concern worldwide, it is a major public health problem in a large proportion of developing countries. Outbreaks often occur seasonally but are amplified during periods of above-normal rainfall in areas of poor sanitation. Extreme climate conditions, such as flooding associated with severe storms and natural disasters such as hurricanes, typhoons, or earthquakes, can disrupt water systems – exposing drinking water to waste water and other effluents – thus increasing the risk of cholera activity and other water-borne infections. Previous works^12,51,52^ have shown links between *El Niño* events, flooding, and cholera outbreaks in various parts of the world. We examine and illustrate enhanced patterns of cholera activity in Tanzania during the warm ENSO event of 2015–2016. Tanzania experiences *Vibrio cholerae* serotype O1 Ogawa, the dominant cholera serotype causing severe diarrheal disorders endemic in most areas of Asia, Sub-Saharan Africa, and South America^53^. Most infections result in mild cases or no sickness at all, although the bacteria may incubate in the gut for 7 to 14 days. When treated rapidly, most people who contract cholera are cured. In the absence of treatment, mortality rates range between 50% and 70%.

Between April 2015–March 2016, cholera was reported (through ProMED) in Tanzania countrywide (Fig. 4e), but no cases were reported in the previous year (April 2014–March 2015) (Fig. S4). The reports were most frequent during and after October 2015 with the highest number of cases reported in December 2015. This was consistent with WHO surveillance reports^54,55^ which showed cholera peaked around October 2015 followed by a short decline until mid-December 2015 when new cases increased again and remained high until about March 2016. When we examined rainfall accumulations in locations with reported cases during November 2015 to January 2016, we found higher-than-normal rainfall during this period with a mean of 280.44 mm above normal (Fig. 4f). These rainfall shifts from normal in locations where cholera cases were reported were continuously observed from September 2015 up to March 2016. Such persistent wet conditions potentially favor cholera propagation when drainage systems become contaminated by effluent.

Using WHO annual cholera case data to represent the disease intensity over the years 2000 to 2017, we observed that the number of cases in 2015 was second highest in the study period, behind 2006 cholera epidemic (Fig. 4g). During *El Niño* years, the disease intensity was higher than in neutral years, i.e., those without an ENSO event (Fig. 4h). The mean disease intensity during *El Niño* years was 2.71% higher than neutral years, and the 2015 intensity was highest among those during *El Niño* years. The number of cases in 2016 was also relatively high, almost as high as 2015. As previously noted, the disease annual counts were based on calendar year, whereas *El Niño* periods span April to March of the following year, and some of the disease data is only available at annual scale. A WHO report^54^ showed that the majority of the cases in 2016 occurred between January to April. Therefore, the high number of cases in 2016 is associated with higher-than-normal precipitation that lasted up to March 2016 (Fig. 2b) which is a lagged response to the *El Niño* event. Multivariate regression further supports the association between cholera cases and higher-than-normal rainfall conditions (Table 3). We found that annual cholera cases were proportionally associated with rainfall anomaly (*P* < 0.05), which means that cholera cases increase as rainfall increases beyond the normal values. We did not find any significant association between annual cholera cases and LST anomaly (Table 3) even though LST conditions were cooler than normal during this period as would be expected (Fig. 1c,d).

#### Dengue in Brazil and Southeast Asia

Dengue fever is a painful, debilitating mosquito-borne disease caused by any one of four related viruses or serotypes transmitted by mosquitoes. It is estimated that ~400 million people are infected yearly, with ~96 million cases resulting in severe illness. The dengue virus is transmitted between people by two species of mosquito vectors; *Aedes aegypti* and *Aedes albopictus*. It is a predominantly tropical disease affecting populations in many areas of the global tropics (Asia, Pacific Islands, Central and South America, and Africa), the region between 30° North and 20° South. Episodic epidemics of dengue have been associated with ENSO in many regions and countries^56–59^. In regions of ENSO teleconnections, persistence of drought and warmer or above-normal temperature conditions affects the dynamics of dengue transmission. Warmer temperatures have several effects on the vector life-cycle and habitats including shortening the maturation period from larva to adult, and increasing biting frequency and hence the propensity to transmit the virus. In addition, the extrinsic incubation period (EIP) is shortened at higher temperatures, thus potentially increasing the proportion of mosquitoes that become infectious at a given time. Dengue epidemics worldwide occur in densely populated urban areas where there is coincidence of large numbers *Aedes aegypti* and *Aedes albopictus* vectors and large numbers of people with no immunity to one of the four virus types. In such densely populated settings, the probability of human–vector contact is very high and appropriate climatic conditions sets the stage for explosive outbreaks. Brazil and Southeast Asia provide good examples of the association of dengue activity during the 2015–2016 *El Niño* event.

Using ProMED reports, we mapped the locations where dengue has been reported since 2000 in Brazil and Southeast Asia (Fig. 4i,m). As dengue is endemic in these areas, the reported cases occurred throughout the regions during the 2015/2016 *El Niño* event. In Brazil, we found that the rainfall distribution shifted to the left of the normal rainfall distribution (towards below-normal values) for locations where dengue was reported between October–December 2015 (Fig. 4j), a period where the disease incidences typically starts to increase. The mean rainfall anomaly during this period and locations was −124.30 mm below normal. This shift to lower-than-normal rainfall was continuously observed until approximately March 2016. We also observed a shift in rainfall towards dry conditions in Southeast Asia during September–November 2015 (Fig. 4n), although the shift was relatively small (mean rainfall of −36.37 mm below normal) and the dry conditions did not persist as long in this region.

As a proxy for disease intensity we used annual dengue cases in Brazil and the number of reports per year (through ProMED) in Southeast Asia. After detrending the time series, dengue cases in Brazil during 2015 was observed to be at the highest, followed by year 2016 (Fig. 4k). The number of cases during *El Niño* years was 2.90% higher than neutral years (Fig. 4l). In Southeast Asia, however, dengue disease intensity during 2015 was relatively low despite forming a local peak (Fig. 4o). Nevertheless, the 2015 intensity was still 1.61% higher than the mean intensity during neutral years (Fig. 4p). In general, we observed that the mean disease intensity during El Niño years was similar to the mean during neutral years (Fig. 4p). Multivariate regression for dengue annual intensity in these two regions indicated that dengue was significantly associated with LST anomaly but not with rainfall anomaly (Table 3). Higher annual dengue intensity was proportionately associated with above-normal LST. Similarly, we observed a shift in LST towards higher-than-normal values in locations with reported dengue cases in both Brazil and Southeast Asia (Supplementary Fig. S8).

## Discussion

The 2015/2016 ENSO event triggered extreme rainfall, drought, and temperature anomaly patterns as we have shown. We observed above-normal rainfall in some regions which not only tended to drive down LSTs (Fig. 1d) because of the shielding effect from the sun of increased cloud cover, but also fostered rapid and persistent vegetation development as shown by regions of positive normalized difference vegetation index (NDVI) anomalies (Supplementary Fig. S3). On the other hand, below-normal rainfall and reduced cloud cover were associated with above-normal LSTs, below-normal NDVI, and drought conditions. Persistence in these ENSO-linked extreme climate conditions provided suitable conditions for disease transmission worldwide during the May 2015–April 2016 period. Disease mapping during this period indicated the tendency for outbreak locations to cluster in ENSO teleconnected regions (Fig. 2a).

In the Southeast Asia region and Brazil, we observed dengue fever outbreaks during the 2015/2016 ENSO event. We simultaneously detected severe and persistent drought conditions throughout the year as indicated by anomalously low precipitation (Figs 1c and 2b). Studies have suggested that associations between severe drought and (1) increased water storage around houses leading to elevated *Aedes aegypti* and *Aedes albopictus* mosquito populations and (2) elevated ambient air temperatures which reduce the EIP for dengue virus in these vectors^31^ increase vectorial capacity and transmission risk. Therefore, the ENSO-induced drought conditions in Southeast Asia and Brazil may contribute to the increase in dengue fever and chikungunya fever outbreaks.

In both the United States and Tanzania, we observed comparatively smaller positive shifts in rainfall during 2015/2016 El Niño period (Fig. 4). Unlike in the 2010–2012 El Niño in the United States and the 2006–2007 El Niño period in East Africa where large shifts from long-term norms created disease outbreak conditions^7^, the observations from the 2015–2016 ENSO period indicate that smaller shifts in climate variables during particular seasons may be sufficient to create ecological conditions for disease vectors and pathogens to emerge and propagate disease outbreaks.

Our analysis indicates that disease activity intensity in some areas was 2.5–28% higher during years with El Niño events than during neutral years. In southeast Asia, dengue intensity during the strong 2015/2016 El Niño was not as high (Fig. 4p), for example, compared to the 1997/1998 event, yet it was still an increase from the previous and following years (Fig. 4o) – forming a local maximum between the years. In three ENSO-teleconnected areas we studied (United States, Tanzania, and Brazil), the disease intensity during the 2015/2016 year was higher than the mean across neutral years as well as the mean across El Niño years. These results add quantifiable evidence to the abundant historical evidence that extreme and highly variable weather conditions and resultant ecological perturbations are closely associated with an elevated risk of disease transmission. These disease events are often proximate outcomes of ecologically enhanced disease-vector population dynamics. They are indirectly facilitated by ENSO-driven ecological processes and with proximate changes in land-use or agricultural practices^60^.

Despite the many advances that have been made in recent years with regard to collecting disease outbreak data, several gaps remain including (1) disease outbreak records are limited and only few^61,62^ are georeferenced – with most epidemiological data available aggregated at larger administrative level (i.e. provincial or national data), therefore there is no precise location of where disease cases were infected, (2) disease outbreaks/activity are not consistently gathered and recorded which leads to biased reporting, (3) there is need for improved and timely reporting of verifiable and confirmed disease outbreaks particularly by government agencies, and (4) there is need to create baseline quantifiable data on disease outbreaks in order to make assessments with regard to climate perturbations. These shortcomings complicate quantifying relationships between climate/weather variability and disease outbreaks. As regional weather anomalies are projected to increase in frequency and severity under global warming scenarios, resulting in more extreme El Niño and La Niña events^63^, improvements in reporting can improve response and prevention measures by better targeting of resources in outbreak regions. In addition to direct effects of temperature and precipitation on infected individuals, outbreaks can exert socio-economic burden on affected regions, including costs to public health systems, costs of mitigation and control, and disruptions of travel, trade, and tourism revenue which acutely distress already fragile economies of small island nation states highly dependent on these industries^64^. Global satellite-based observation systems monitoring key climate variables combined with seasonal forecasts can be regionally calibrated to identify periods of elevated disease risk, and through early warning, reduce impacts of ecologically coupled diseases and help mitigate risks of global spread of preventable and controllable diseases.

## Methods

### ENSO and Sea Surface Temperature (SST) Data

We used the NINO 3.4 SST ENSO index, obtained from the National Oceanic and Atmospheric Administration (NOAA)’s National Center for Climate Prediction on-line archives at, http://www.cpc.ncep.noaa.gov/data/indices/sstoi.indices. The warm and cold periods of ENSO events were determined using the Oceanic Niño Index (ONI) threshold of +/− 0.5 °C based on centered 30-year base periods updated every 5 years^65^. The ONI is a 3-month running mean of Extended Reconstructed Sea Surface Temperature (ERSST) Version 4 (v4) SST anomalies in the Niño 3.4 region (5°N–5°S, 120°–170°W, see Supplementary Fig. S11).

For monthly SST, we used the Optimum Interpolation (OI) SST version 2 dataset produced by NOAA (https://www.ncdc.noaa.gov/oisst). The SST was produced weekly using both *in situ* and satellite data and merged to create a monthly data set^66^. This dataset is available from 1981 to present with 1° × 1° spatial resolution.

### Rainfall, Land Surface Temperature, and Vegetation Index Data

Rainfall data for Brazil and Southeast Asia were obtained from NASA’s Global Precipitation Climatology Project (GPCP) dataset^67,68^ and NOAA’s Climate Prediction Center (CPC) unified (UNI) datasets^69,70^. GPCP is a monthly dataset from 1979 to present  and has 2.5° × 2.5° spatial resolution^68^; while CPC UNI is also a monthly dataset (1979–present) with 0.5° × 0.5° spatial resolution^69^. For Tanzania, we used the daily African Rainfall Climatology (ARC) dataset from the NOAA – CPC archives^71,72^. The dataset is available over Africa at 0.1° × 0.1° spatial resolution from 1983 to present . Both the GPCP and ARC datasets are produced using a combination of rainfall gauge measurements and satellites to produce the gridded rainfall estimates^68,71,72^. Rainfall data for the United States were obtained from NOAA’s National Stage IV dataset^73,74^, which is available as hourly, 6-hourly, and daily datasets with 4 km resolution. Here, rainfall is estimated based on rainfall gauge and radar data over the continental United States.

Both land surface temperature (LST) and the normalized difference vegetation index (NDVI) datasets are derived from NASA’s Earth Observing System Moderate Resolution Imaging Spectroradiometer (MODIS) instrument aboard the Terra (EOS AM-1) spacecraft. We used the MODIS global monthly Climate Modeling Grid (CMG) products with a spatial resolution of 0.05° × 0.05° (~5.5 × 5.5 km). Detailed explanations on these data sets and associated references are provided in the Supplementary Information text.

### Disease Outbreak Data

We monitored, systematically recorded, and georeferenced outbreaks of selected diseases around the world from September 2014 to April 2016 by searching online reports of the Program for Monitoring Emerging Diseases (ProMED), Pan-American Health Organization (PAHO) online country reports, and weekly summaries of disease outbreaks reported by the Department of Defense Armed Forces Health Surveillance Branch gathered from various sources^27,36,75^. These reports were compared for commonalities and merged as necessary to create a rolling disease database during the 2015–2016 ENSO event. Such disease outbreak reports provide key material for developing climate-based early warning of emerging disease outbreaks worldwide. The following diseases were selected for monitoring for outbreaks during this ENSO event: chikungunya, cholera, dengue, hantavirus, malaria, plague, respiratory illness, Rift Valley fever, Ross River fever, St. Louis encephalitis, tularemia, and Zika. A number of these diseases have been shown to be associated with ENSO events^7,9,10,12,13^. Georeferencing provides a spatial anchor to determine where and when a particular outbreak has occurred and what type of weather and/or ecological anomaly is associated with the outbreak. As in many cases of such passive surveillance systems, it is difficult to determine the precise numbers of individuals or populations affected by an outbreak because of under-recognition of diseases or non-specific symptoms (e.g., cases of hemorrhagic fevers in Sudan and South Sudan during this period), delays in reporting due to lack of adequate laboratory support, and large variations in reporting systems among countries^76^. We therefore have focused on the geographic mapping of disease outbreaks to discern timing and patterns in relation to weather anomalies rather than the number of people affected (such numbers where provided should be considered rough estimates). Our overarching purpose is to begin to create standardized disease outbreak databases at a global scale, which can be used to study and characterize the relationships between climate and/or weather variability and outbreak patterns of select diseases.

In addition, we compiled a list of outbreak reports from ProMED of selected diseases (dengue for Southeast Asia, hantavirus and plague for United States) to document the number of times a particular disease was reported in a given country. The numbers of reports were then aggregated to produce monthly and annual data for the period 2002 to 2016. We used this “number of reports” as an indicator or proxy for the disease outbreak intensity. We also obtained annual dengue data for Brazil from PAHO between 2002–2016^75^ and annual cholera data for Tanzania from WHO Weekly Epidemiological Record (WER)^42^. The annual “number of reports” and annual cases were de-trended to remove possible bias due to enhancements in reporting systems and/or practices across the years. The trend over time was estimated using a linear regression with year as the independent variable.

### Analysis

Weather and environmental anomalies were calculated by first generating the long-term mean, or *climatology*, of the monthly rainfall (accumulation), LSTs, and NDVI for each of the 12 months, across the years that data were available. Monthly anomalies were then calculated by subtracting the corresponding month’s long-term mean from the current month’s value. For deriving anomalies spanning a number of months, for instance a specified interval or a season, the long-term mean was calculated by first averaging the monthly data (summing in case for rainfall) for those specific months in each year of the data set and then calculating the average across all available years. Details on these methods are provided in the Supplementary Information text.

For the anomaly distributions shown in Fig. 4(b,f,j,n), we selected a 3-month period for each region when El Niño has the highest impact (http://iri.columbia.edu/wp-content/uploads/2016/05/ElNino_Rainfall.pdf). This period typically coincides with the growing season and also the highest disease transmission risk season. The periods selected are May-July for the United States, October-December for Brazil, November-January for Tanzania, and September-November for Southeast Asia. We then calculated the 3-month anomalies and subsequently extracted the data from the spatial extent of where outbreaks have been reported to occur in that period of time.

In order to assess disease intensity between years with and without ENSO events, we classify the years based on the ONI values. Briefly, ONI is an indicator of ENSO events which is calculated based on Sea Surface Temperature (SST) in the Niño 3.4 region. A running 3-month mean of SST was calculated and then compared to the 30-year average. The ONI value is the difference between these two values. An ONI value higher than +0.5 °C indicates *El Niño* conditions, and conversely, a value less than −0.5 °C indicates *La Niña* conditions. In the analysis, we classified years with ONI ≥ 1 °C as *El Niño* year, ONI ≤ −1 °C as *La Niña* year, and |ONI| < 1 °C as neutral year. We used |1 °C | ONI value as the threshold instead of the more frequently used |0.5 °C| because of the variability in the climate response to ENSO events such that we are only considering strong ENSO events.

The association between ENSO-induced climate anomalies (rainfall and LST) and disease intensity was examined using multivariate regression. As a measure of disease intensity, we used the annual number of reports for hantavirus and plague in the United States, annual number of cholera cases in Tanzania, annual number of dengue cases in Brazil, and annual number of dengue reports for Southeast Asia. Because of the different types of dataset we are using, we first normalize the annual number of reports and cases by calculating the z-score (subtracting the mean and dividing by the standard deviation). These values are then used as the dependent variable. We also calculated the z-score for annual rainfall and LST anomalies, which are then used as independent variables. Year was also included as independent variable to account for trend. Autocorrelation within the independent variable was examined using autocorrelation function plots (Supplementary Fig. S12), in which we did not detect any significant autocorrelations. Collinearity between the independent variables were assessed by calculating the variable inflation factor (VIF) – a factor of how much the coefficient’s standard error would increase if the said covariate were not correlated with the others. A value of 1 indicates that the covariate is orthogonal to the others, and common practice considers VIF of 5 or 10 suggests severe collinearity^77,78^. In our analysis, all VIF values were less than 4. All statistical analysis was performed using R software^79^.

## Electronic supplementary material

Supplementary Information
