# Author Correction: Global disease outbreaks associated with the 2015–2016 El Niño Event

**DOI:** 10.1038/s41598-020-73760-3

**Published:** 2020-10-15

**Authors:** Assaf Anyamba, Jean-Paul Chretien, Seth C. Britch, Radina P. Soebiyanto, Jennifer L. Small, Rikke Jepsen, Brett M. Forshey, Jose L. Sanchez, Ryan D. Smith, Ryan Harris, Compton J. Tucker, William B. Karesh, Kenneth J. Linthicum

**Affiliations:** 1 Universities Space Research Association, Columbia, MD USA; 2 NASA Goddard Space Flight Center, Biospheric Sciences Laboratory, Greenbelt, MD USA; 3 Department of Defense, Armed Forces Health Surveillance Branch, Silver Spring, MD USA; 4 USDA-Agricultural Research Service Center for Medical, Agricultural, and Veterinary Entomology, Gainesville, FL USA; 5 Science Systems and Applications, Inc., Lanham, MD USA; 6 Cherokee Nation Technology Solutions, Silver Spring, MD USA; 7 United States Air Force, 14th Weather Squadron - DoD Climate Services, Asheville, NC USA; 8 EcoHealth Alliance, New York, NY USA; 9 Present Address: National Center for Medical Intelligence, Fort Detrick, MD USA; 10 Present Address: Interstate Commission on the Potomac River Basin, Rockville, MD USA

Correction to: *Scientific Reports* 10.1038/s41598-018-38034-z, published online 13 February 2019

The original version of the Article contained several errors.

An affiliation for Brett M. Forshey was omitted and is now listed as Affiliation 6.

Furthermore, in the HTML version of this Article Brett M. Forshey was incorrectly affiliated with ‘United States Air Force, 14th Weather Squadron - DoD Climate Services, Asheville, North Carolina, USA’. The correct affiliations for Brett M. Forshey are listed below.

Department of Defense, Armed Forces Health Surveillance Branch, Silver Spring, Maryland, USA

Cherokee Nation Technology Solutions, Silver Spring, Maryland, USA

Additionally, in the legend of Figure 1,

“(c) May-July 2016 cumulative rainfall anomalies during the ENSO early phase, and”

now reads:

“(c) October - December 2015 cumulative rainfall anomalies towards the ENSO peak phase and”

In the legend of Figure 2,

“(b) and time series profiles of climate variables (a) for each box in (b). Persistence of anomaly conditions of precipitation, land surface temperature, and normalized difference vegetation index in (a) created conditions for the emergence of vectors and outbreaks of diseases for United States, Brazil, Tanzania, and Southeast Asia focal regions in (b)”

now reads:

“(a) and time series profiles of climate variables (b) for each box in (a). Persistence of anomaly conditions of precipitation, land surface temperature, and normalized difference vegetation index in (b) created conditions for the emergence of vectors and outbreaks of diseases for United States, Brazil, Tanzania, and Southeast Asia focal regions in (a)”

Furthermore, in the Results section under the subheading ‘ENSO-induced anomalies in weather and environmental conditions worldwide’,

“This higher-than-normal SST anomaly reached its peak conditions in November 2016 (Fig. 1a right panel and Fig. 1b).”

now reads:

“This higher-than-normal SST anomaly reached its peak conditions in December 2015-February 2016 (Fig. 1a right panel and Fig. 1b).”

And,

“Many areas of South and Central America and the Caribbean Islands had coincident outbreaks of Zika, dengue fever, and chikungunya in 2015–2016 (Fig. 2a and Supplementary Fig. S4).”

now reads:

“Many areas of South and Central America and the Caribbean Islands had coincident outbreaks of Zika, dengue fever, and chikungunya in 2015–2016 (Fig. 2a and Supplementary Fig. S3).”

And,

“This drought was a result of the shift in the center of maximum precipitation from the western Pacific equator towards the eastern Pacific under El Niño conditions (Supplementary Fig. S1C).”

now reads:

“This drought was a result of the shift in the center of maximum precipitation from the western Pacific equator towards the eastern Pacific under El Niño conditions (Supplementary Fig. S2).”

Lastly, in the Discussion,

“Disease mapping during this period indicated the tendency for outbreak locations to cluster in ENSO teleconnected regions (Fig. 2b).”

now reads:

“Disease mapping during this period indicated the tendency for outbreak locations to cluster in ENSO teleconnected regions (Fig. 2a).”

And,

“In both the United States and Tanzania, we observed comparatively smaller positive shifts in rainfall during 2015/2016 El Niño period (Fig. 3).”

now reads:

“In both the United States and Tanzania, we observed comparatively smaller positive shifts in rainfall during 2015/2016 El Niño period (Fig. 4).”

These errors have now been corrected in the PDF and HTML versions of the Article, and in the accompanying Supplementary Information.

