# Supplementary material for: Global Disease Outbreaks Associated with the 2015–2016 El Niño Event
Source: Sci Rep. 2019 Feb 13;9:1930. doi: 10.1038/s41598-018-38034-z (PMC6374399; doi:10.1038/s41598-018-38034-z)
Supplement: Supplementary file 1 — Supplementary Information [file 41598_2018_38034_MOESM1_ESM.pdf]

Supplementary Information

Global Disease Outbreaks Associated with the 2015-2016 El Niño Event

Assaf Anyamba[1,2*], Jean-Paul Chretien[3,9], Seth C. Britch[4], Radina P. Soebiyanto[1,2], Jennifer L. Small[2,5], Rikke Jepsen[2,5,10], Brett M. Forshey[3,6], Jose L. Sanchez[3], Ryan D. Smith[7], Ryan Harris[7], Compton J. Tucker[2], William B. Karesh[8], Kenneth J. Linthicum[4]

1 Universities Space Research Association, Columbia, Maryland, USA.
2 NASA Goddard Space flight center, Biospheric Sciences Laboratory, Greenbelt, Maryland, USA.
3 Department of Defense, Armed forces Health Surveillance Branch, Silver Spring, Maryland, USA.
4 USDA-Agricultural Research Service Center for Medical, Agricultural, and Veterinary Entomology, Gainesville, Florida, USA.
5 Science Systems and Applications, inc., Lanham, Maryland, USA.
6 Cherokee Nation Technology Solutions, Silver Spring, Maryland.
7 United States Air Force, 14th Weather Squadron - DoD Climate Services, Asheville, North Carolina, USA.
8 EcoHealth Alliance, New York, New York, USA.
9 Present address: National Center for Medical intelligence, Fort Detrick, Maryland, USA.
10 Present address: Interstate Commission on the Potomac River Basin, Rockville, Maryland, USA.

*Corresponding author
Email: assaf.anyamba@nasa.gov

This PDF file includes:

Materials, Methods, and Data Sources.
Figs. S1 to S11.
References (1-14).

1. Land Surface Temperature (LST) and Normalized Difference Vegetation Index (NDVI)

Land surface temperature (LST) is a key parameter in land surface processes. The difference between LST and surface air temperature drives energy exchange at the planetary boundary layer. Changes in LST can induce convection at the boundary layer and influence air temperature, surface winds, cloudiness, and precipitation. LST is used in global climate models for heat budget and boundary layer energy balance calculations, and has also proved useful for agricultural applications in estimating crop water demands and drought severity assessments[1]. When plants encounter water deficit, transpiration is reduced causing leaf temperature (which is equal to LST for dense vegetation) to rise. On the other hand, when water is not limited, transpiration will result in cooling of the leaf temperature relative to air temperature. We used LST to infer temperature conditions on the land surface especially in vegetated areas, which serve as potential vector emergence sites during the 2015-2016 El Niño period [2]. In this study, we used the global monthly 0.05° MOD11C3 data set (https://lpdaac.usgs.gov/dataset_discovery/modis/modis_products_table/mod11c3). MODIS LST is derived from daytime and nighttime thermal infrared measurements in bands 31 (10.8-11.3 nm) and 32 (11.8-12.3 nm) using the day/night LST algorithm described in[2]. Cloud screening is performed using the MODIS cloud mask product (MOD35_L2, https://modaps.modaps.eosdis.nasa.gov/services/about/products/c6/MOD35_L2.html) prior to the LST calculation. Estimated accuracy for the MODIS LST product is 1° Kelvin for land cover types with known emissivity.

The NDVI and similar vegetation indices are widely used to infer the photosynthetic capacity of vegetation and are used as a land surface input in various weather, climate, biogeochemical, and hydrological models[3]. Applications of NDVI are numerous and varied and include agricultural monitoring, famine early warning, ecological monitoring for habitat indicative of pest and arthropod vector emergence and survival, and determination of land use and land cover changes, among others[4]. The NDVI is simply the ratio of the difference between the near-infrared and red reflectance to their sum; since green leaves with dense chlorophyll are more reflective in the near-infrared wavelengths than in the visible, this ratio is higher (approaching one) for healthy green vegetation and lower (approaching zero) for stressed vegetation[4]. MODIS NDVI data are derived from the red and near-infrared bands, centered at 648 nm and 848 nm, respectively. The band reflectance data are atmospherically corrected and masked for cloud, cloud shadow, and aerosol contamination[5]. In this study we used the global monthly 0.05° MOD13C2 NDVI; this product has been aggregated from 250 m MODIS NDVI as described above.

For this study we were interested in weather extremes and their impact on disease outbreaks. It has been shown that there is a close relationship between the seasonal trace of green vegetation development, as measured by NDVI, and breeding and upsurge patterns of particular insects, such as locusts and mosquitoes[6–9]. Widespread heavy rains result in rapid vegetation development that provides ideal habitat for immature and adult mosquito vector populations to increase and thrive, significantly elevating the risk of disease outbreaks[10,11]. Emergent vegetation is also associated increase in rodent populations in semi-arid areas as vegetative growth provides food resources for rodents and thus an increase in the transmission risk of plague and

hantavirus[12].

In general, departures in NDVI and LST are associated with various disease ecologies. Many key arthropod disease vectors and arthropod-borne pathogens emerge and persist when conditions depart from average in various landscapes. This relationship exists because these departures in NDVI and LST are driven to a large extent by variations in rainfall influencing landscape patterns[2]. To illustrate the strong relationships among LST, NDVI, and rainfall we used the Global Precipitation Climatology Project (GPCP) *Global 1° Monitoring Product*, available at ftp://ftp-anon.dwd.de/pub/data/gpcc/html/monitoring_ download.html, to compare rainfall totals for each region and to the monthly and seasonal means. We aggregated the monthly GPCP data to seasonal sums, calculated the 1979-2015 average seasonal sums, and finally the seasonal rainfall anomalies for each season of interest. Seasonal rainfall totals, means, and percent anomalies are presented by region in **Table S1**. Note that the seasons in each region are selected based on the periods when El Niño has the highest impact (see Methods section in the Main Text).

2. Precipitation Anomaly Forecast

As mentioned in the main manuscript, we used precipitation anomaly forecasts to calibrate disease transmission risk as part of the ENSO-induced disease monitoring effort orchestrated by NASA, USDA, and the DoD. Precipitation anomaly forecasts are derived from the anomalous precipitation rate forecast output from the North American Multi-Model Ensemble (NMME)[13]. The data are interpolated from the native 1° x 1° scale to a finer 0.5° x 0.5° resolution, and then extrapolated in time from units of mm s^{-1} to mm $month^{-1}$ (effectively mm of precipitation for a given month). Extrapolation is based on a constant anomalous precipitation rate across the total number of days in the calendar year and month for which the forecast is valid (here October, November, or December 2015) accounting for leap days. For forecasts at seasonal time scales (e.g., October – December), a sum is taken of the three individual monthly precipitation forecasts. Results of such forecast products are shown in **Fig. S1**. Regions anomalous in either direction, that is, experiencing drought or excessive rainfall and possibly flooding, are determined to be areas of potential disease emergence and used in early warning alerts.

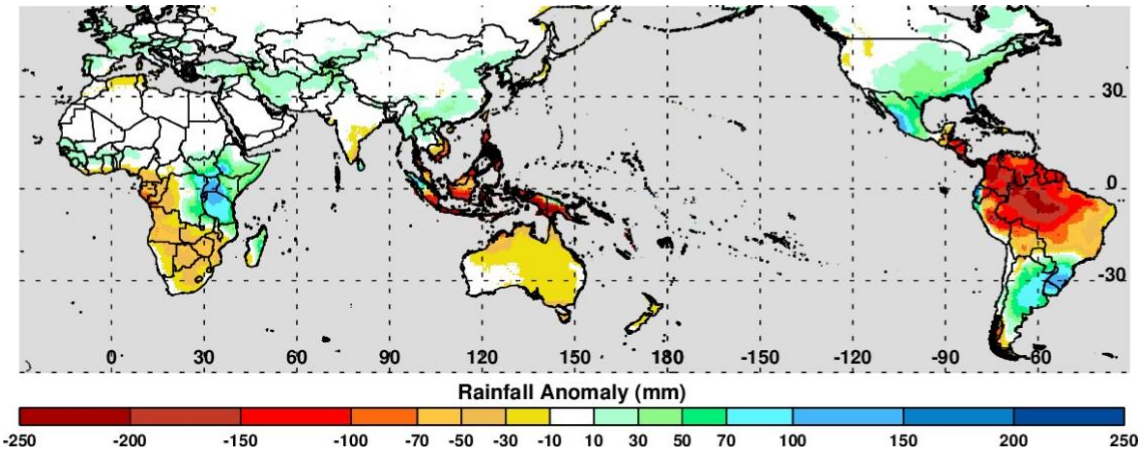

Fig. S1. Forecast cumulative rainfall anomalies for October- - December 2015. Note regions of forecast above-normal rainfall in Eastern Africa, Southern Brazil/Argentina, and southern US/Mexico, and below-normal rainfall including northern South America, Southern Africa, Southeast Asia, and Australia. This figure was created using Interactive Data Language (IDL) software (version 8.6.0) (www.harrisgeospatial.com/SoftwareTechnology/IDL.aspx).

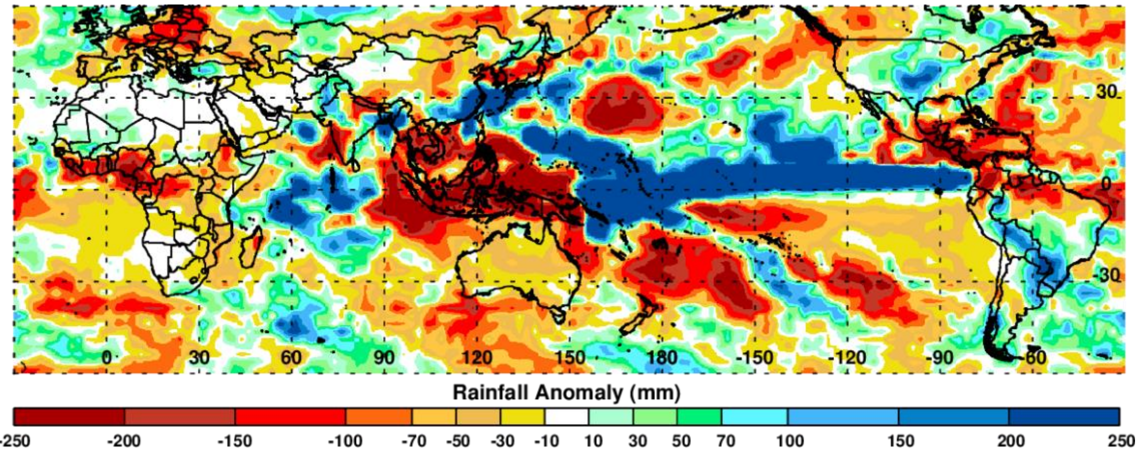

Fig. S2. Observed cumulative rainfall anomalies for May, June, July, August 2015. This figure was created using Interactive Data Language (IDL) software (version 8.6.0) (www.harrisgeospatial.com/SoftwareTechnology/IDL.aspx).

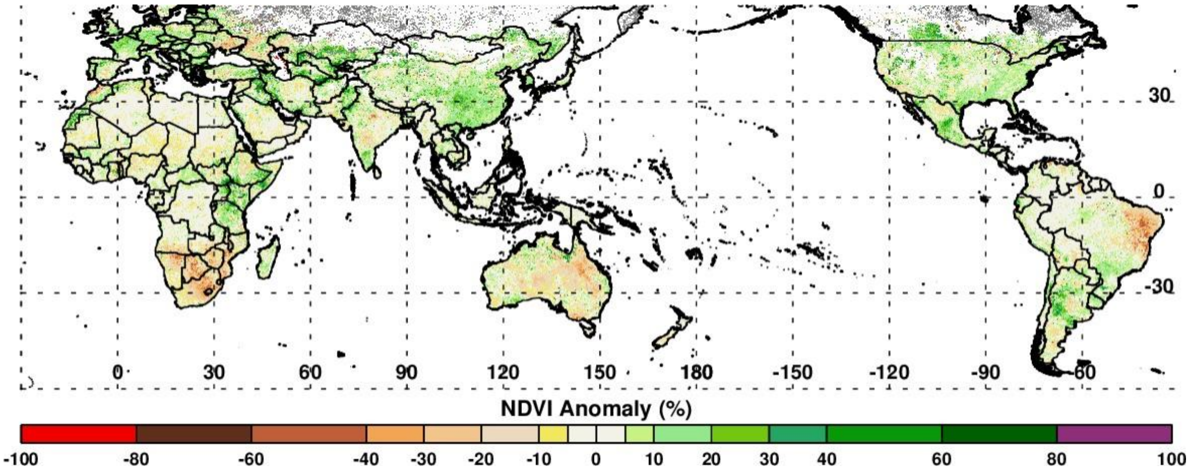

Fig. S3. Observed normalized difference vegetation index anomalies for December 2015. The patterns of vegetation anomalies here agree with the forecast anomalies and observed precipitation anomalies shown in Fig. S1 and Fig. S3 above. This figure was created using Interactive Data Language (IDL) software (version 8.6.0) (www.harrisgeospatial.com/SoftwareTechnology/IDL.aspx).

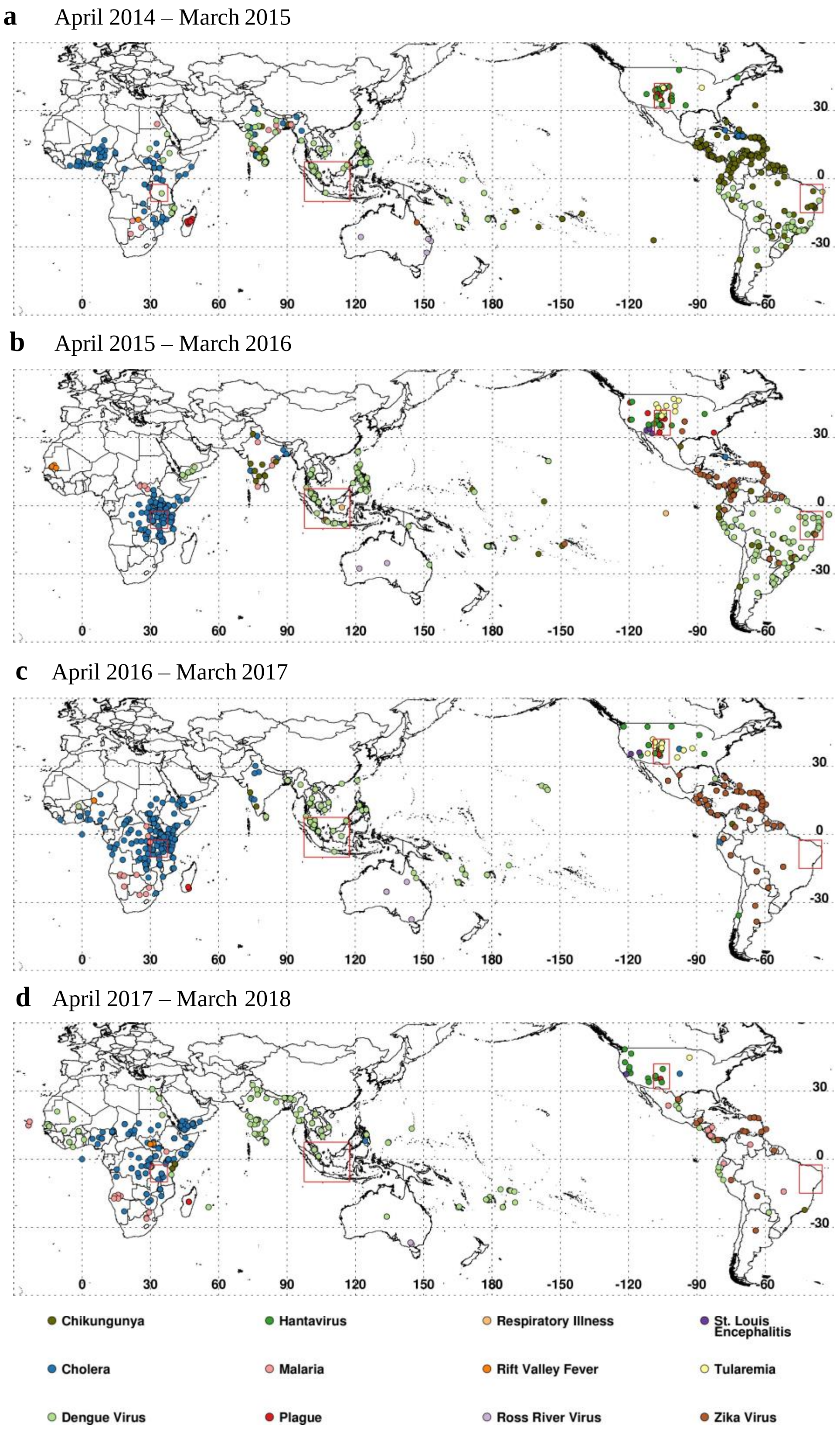
a April 2014 – March 2015
b April 2015 – March 2016
c April 2016 – March 2017
d April 2017 – March 2018
30
0
-30
0 30 60 90 120 150 180 -150 -120 -90 -60
Chikungunya
Hantavirus
Respiratory Illness
St. Louis Encephalitis
Cholera
Malaria
Rift Valley Fever
Tularemia
Dengue Virus
Plague
Ross River Virus
Zika Virus

Fig. S4. Climate-sensitive diseases that were reported through ProMED between 2014 to 2018. This figure was created using Interactive Data Language (IDL) software (version 8.6.0) (www.harrisgeospatial.com/SoftwareTechnology/IDL.aspx)

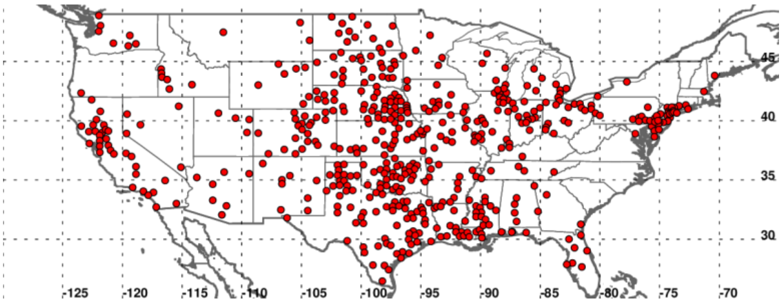

Fig. S5. West Nile fever activity in the US in 2015. Note the clustering in central and southern US and California regions of maximum precipitation departures in shown in S3. This figure was created using Interactive Data Language (IDL) software (version 8.6.0) (www.harrisgeospatial.com/SoftwareTechnology/IDL.aspx).

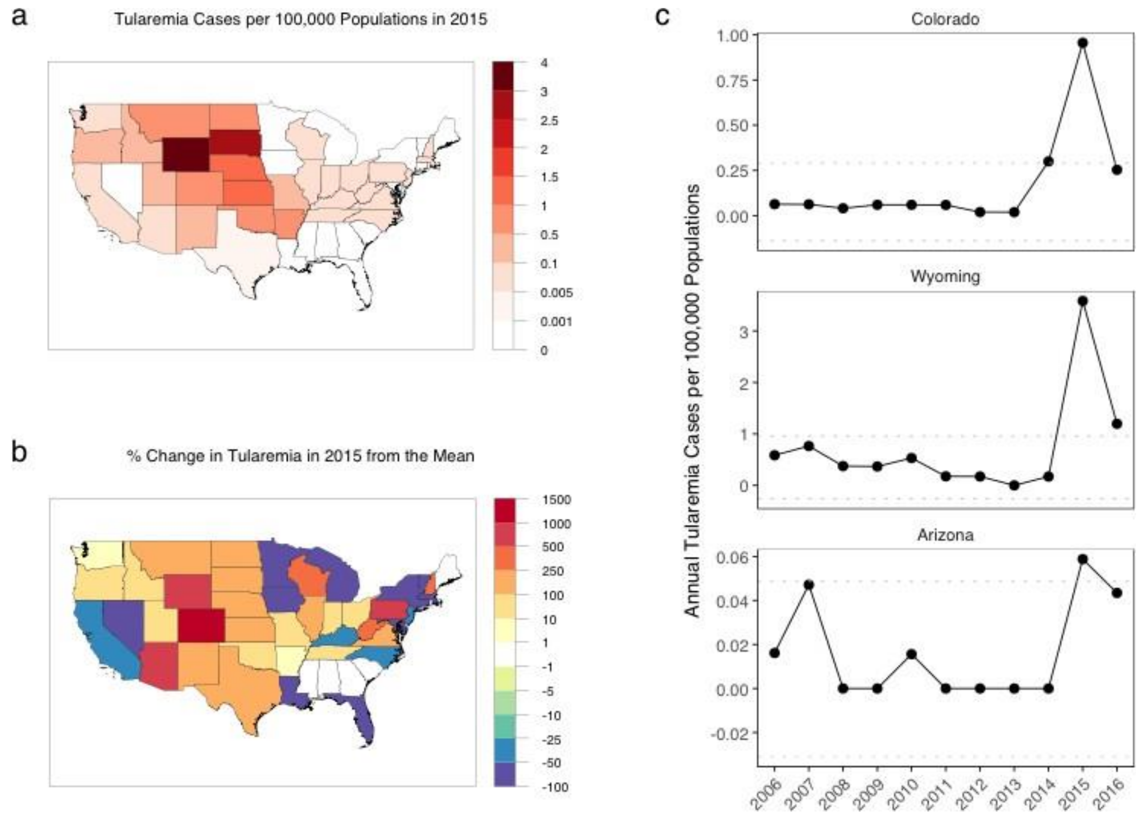

Fig. S6. Annual Tularemia cases per 100,000 populations. (a) In year 2015. (b) Percent change in Tularemia cases per 100,000 populations from the mean in 2015. (c) Annual cases per 100,000

populations between 2006 – 2016 for the top 3 states with highest increase in 2015 from the mean. Grey dashed lines indicate annual mean ±2.5 standard deviation. Annual Tularemia cases and population by states were obtained, respectively, from the CDC [https://www.cdc.gov/tularemia/statistics/index.html] and US Census [https://www.census.gov/data/tables/2017/demo/popest/nation-total.html#par_textimage_2011805803]. This figure was created using R software (version 3.4.1)[14].

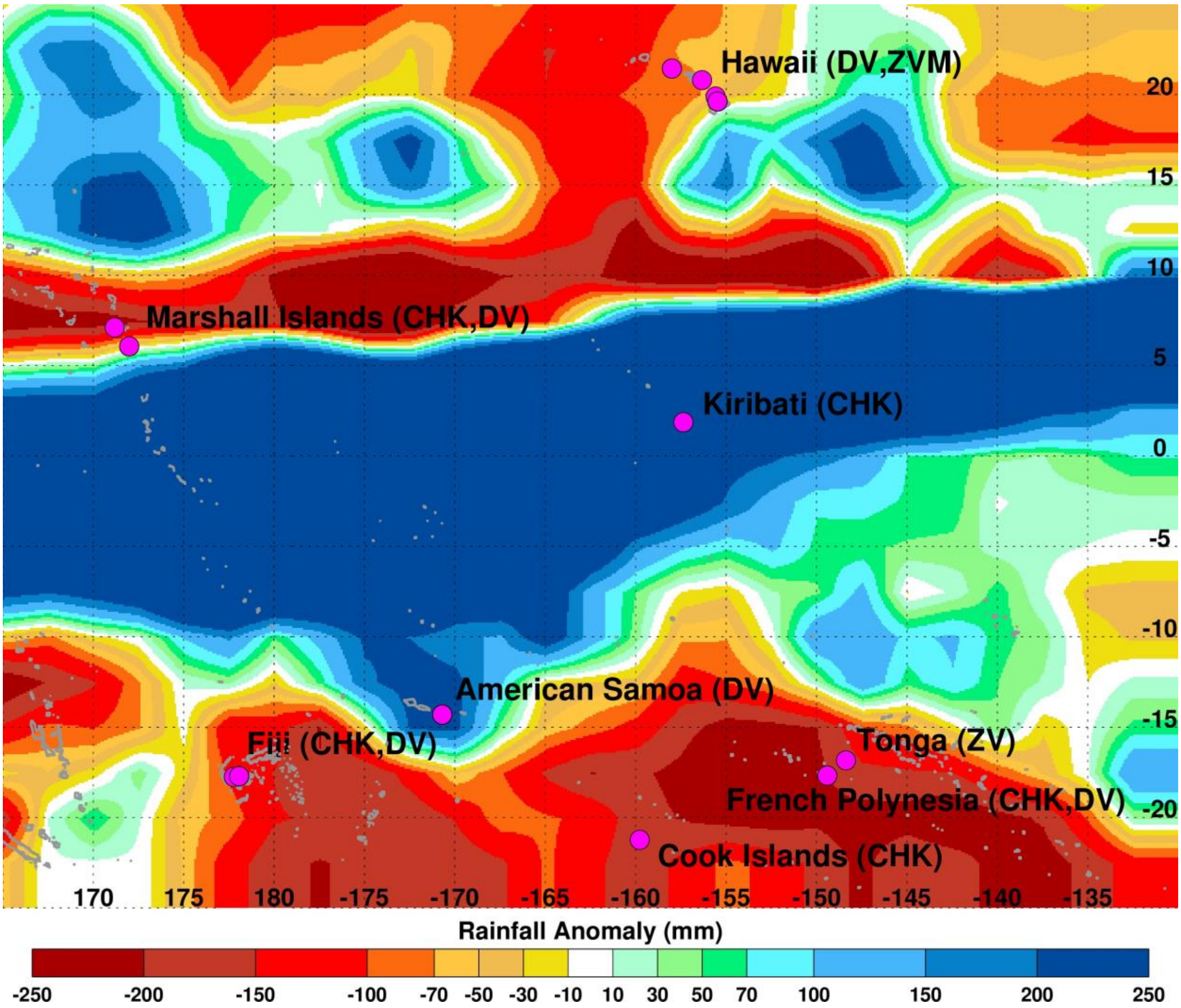

Fig. S7. Observed cumulative rainfall anomalies for October - December 2015 for the Pacific Islands **(**Tonga, Hawaii, America Samoa, Cook Islands, Fiji Islands, French Polynesia, and Marshall Islands) showing the predominance of lower-than-normal conditions or drought in areas of outbreaks of chikungunya (CHK), dengue fever (DV), and Zika (ZV). This figure was created using Interactive Data Language (IDL) software (version 8.6.0) (www.harrisgeospatial.com/SoftwareTechnology/IDL.aspx).

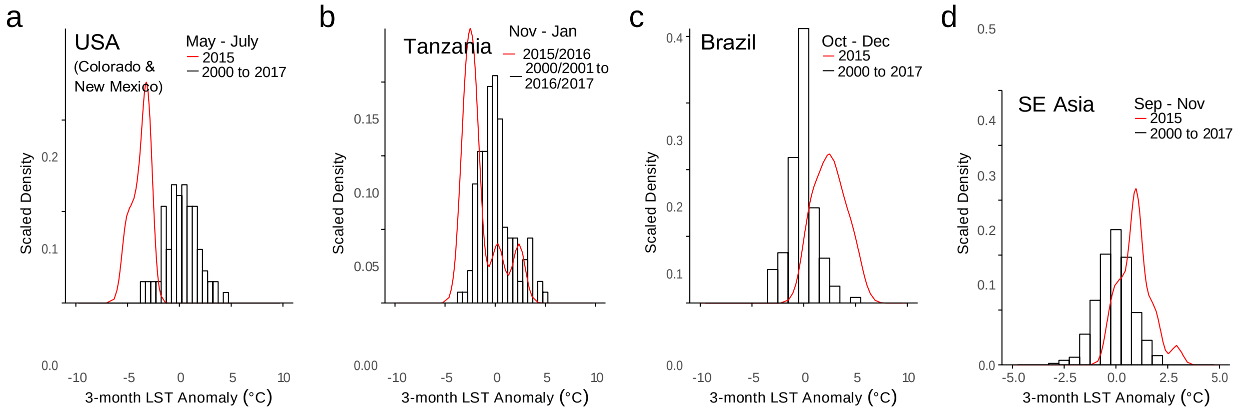

Fig. S8. Land Surface Temperature (LST) distribution of 3-month average anomaly during 2015/2015 El *Niño* year (in red) compared to the same 3-month window in other years (in black). The distributions were generated using those pixels where outbreaks were reported during the specified 3-month period. This figure was created using R software (version 3.4.1)[14].

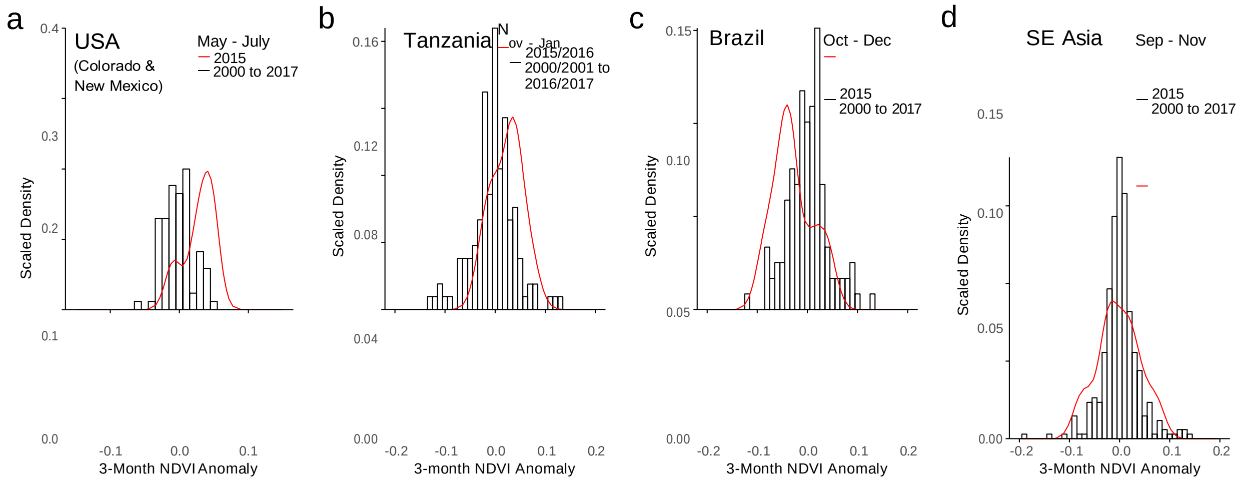

Fig. S9. Normalized Difference Vegetation Index (NDVI) 3-month average anomaly during 2015/2015 El *Niño* year (in red) compared to the same 3-month window in other years (in black). The distributions were generated using those pixels where outbreaks were reported during the specified 3-month period. This figure was created using R software (version 3.4.1)[14].

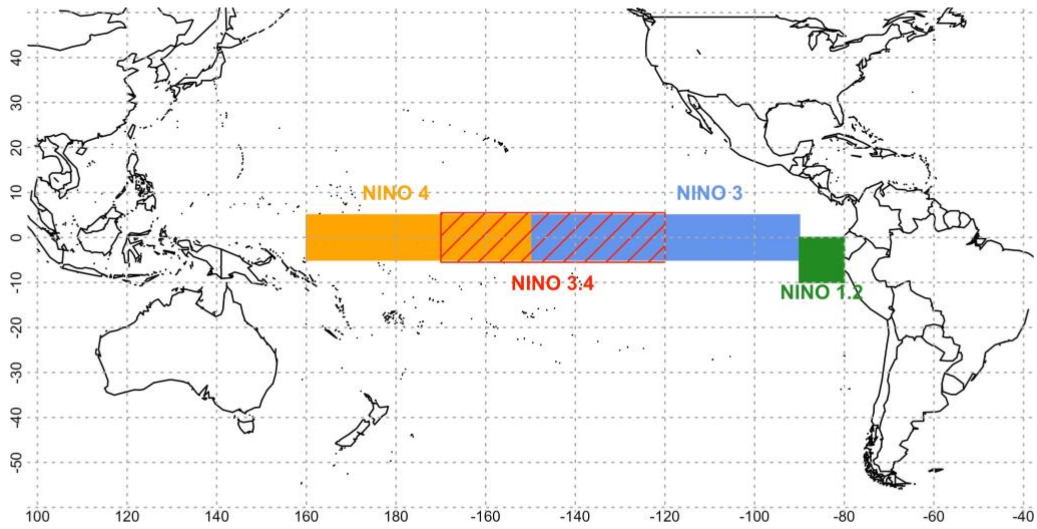

Fig. S10. Niño 3.4 region is in red (5°N – 5°S, 120°W – 170°W); Niño 3 region in orange (5°N – 5°S, 150°W – 90°W); Niño 4 region in blue (5°N – 5°S, 160°E – 150°W); Niño 1.2 region in green (0° – 10°S, 90°W – 80°W. This figure is generated using R software (version 3.4.1)[14].

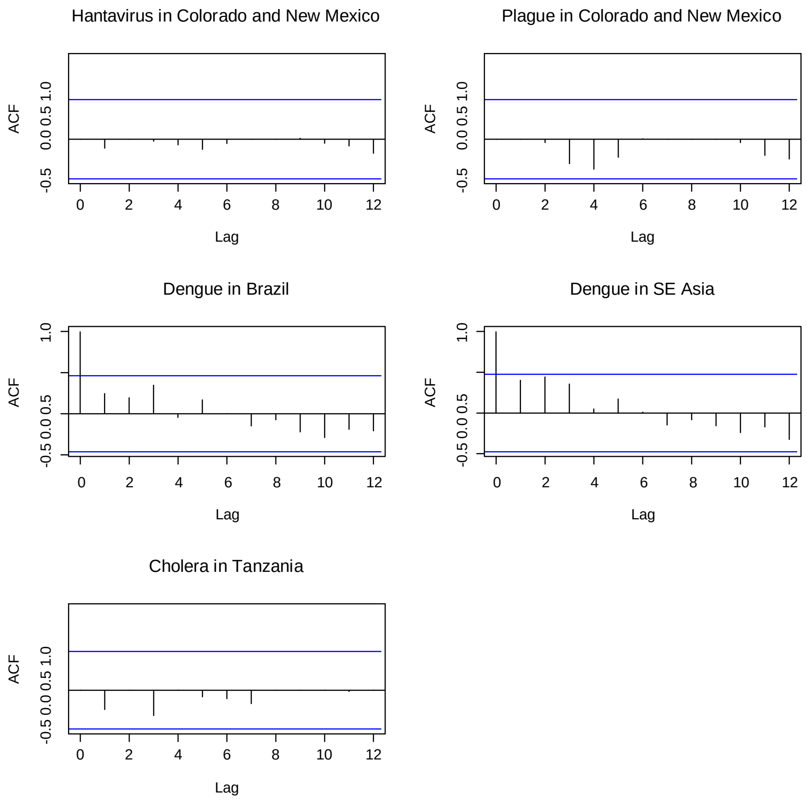

Fig. S11. Autocorrelation Function (ACF) plots for dependent variable (annual disease report or count). This figure was created using R software (version 3.4.1)[14].

References:

1. Wan, Z., Zhang, Y., Zhang, Q. & Li, Z. Validation of the land-surface temperature products retrieved from Terra Moderate Resolution Imaging Spectroradiometer data. *Remote Sens. Environ.* **83,** 163–180 (2002).
2. Anyamba, A. *et al.* Recent Weather Extremes and Impacts on Agricultural Production and Vector-Borne Disease Outbreak Patterns. *PLoS One* **9,** e92538 (2014).
3. Townshend, J. R. G. & Justice, C. O. Towards operational monitoring of terrestrial systems by moderate-resolution remote sensing. *Remote Sens. Environ.* **83,** 351–359 (2002).
4. Tucker, C. J. History of the Use of AVHRR Data for Land Applications. in *Advances in the Use of NOAA AVHRR Data for Land Applications* 1–19 (Springer Netherlands, 1996). doi:10.1007/978-94-009-0203-9_1
5. Karnieli, A. *et al.* Use of NDVI and Land Surface Temperature for Drought Assessment: Merits and Limitations. *J. Clim.* **23,** 618–633 (2010).
6. Tucker, C. J., Hielkema, J. U. & Roffey, J. The potential of satellite remote sensing of ecological conditions for survey and forecasting desert-locust activity. *Int. J. Remote Sens.* **6,** 127–138 (1985).
7. Hielkema, J. U., Roffey, J. & Tucker, C. J. Assessment of ecological conditions associated with the 1980/81 desert locust plague upsurge in West Africa using environmental satellite data. *Int. J. Remote Sens.* **7,** 1609–1622 (1986).
8. Linthicum, K. J., Bailey, C. L., Davies, F. G. & Tucker, C. J. Detection of Rift Valley fever viral activity in Kenya by satellite remote sensing imagery. *Science* **235,** 1656–9 (1987).
9. Linthicum, K. J. *et al.* Application of polar-orbiting, meteorological satellite data to detect flooding of Rift Valley Fever virus vector mosquito habitats in Kenya. *Med. Vet. Entomol.* **4,** 433–8 (1990).
10. Linthicum, K. J., Davies, F. G., Bailey, C. L. & Kairo, A. Mosquito species succession in a dambo in an East African forest. *Mosq. News* **43,** 464–470 (1983).
11. Linthicum, K. J., Davies, F. G., Bailey, C. L. & Kairo, A. Mosquito species encountered in a flooded grassland dambo in Kenya. *Mosq. News* **44,** 228–232 (1984).
12. Eisen, R. J. *et al.* A spatial model of shared risk for plague and hantavirus pulmonary syndrome in the southwestern United States. *Am. J. Trop. Med. Hyg.* **77,** 999–1004 (2007).
13. Kirtman, B. P. *et al.* The North American Multimodel Ensemble: Phase-1 Seasonal-to-Interannual Prediction; Phase-2 toward Developing Intraseasonal Prediction. *Bull. Am. Meteorol. Soc.* **95,** 585–601 (2014).
14. R Core Team. R: A Language and Environment for Statistical Computing. (2017).
